# In Situ Printing of Polylactic Acid/Nanoceramic Filaments for the Repair of Bone Defects Using a Portable 3D Device

**DOI:** 10.1021/acsami.4c05232

**Published:** 2024-07-21

**Authors:** Guilherme Castro Brito, Gustavo Fernandes Sousa, Moises Virgens Santana, André Sales Aguiar Furtado, Millena de Cassia Sousa E Silva, Thiago Ferreira Candido Lima Verde, Renata Barbosa, Tatianny Soares Alves, Luana Marotta Reis Vasconcellos, Leonardo Alvares Sobral Silva, Vicente Galber Freitas Viana, José Figueredo-Silva, Antônio Luiz Martins Maia Filho, Fernanda Roberta Marciano, Anderson Oliveira Lobo

**Affiliations:** † LIMAV−Interdisciplinary Laboratory for Advanced Materials, BioMatLab, Materials Science & Engineering Graduate Program, UFPI−Federal University of Piauí, Teresina 64049-550, Piauí, Brazil; ‡ LAPCON—Laboratory of Polymers and Conjugated Materials, Technology Center CT, Materials Science & Engineering Graduate Program, UFPI−Federal University of Piauí, Teresina 64049-550, Piauí, Brazil; § Institute of Science and Technology, São Paulo State University (UNESP) 777 Eng. Francisco José Longo Avenue, São José dos Campos 12245-000, São Paulo, Brazil; ∥ Postgraduate Program in Materials Engineering, Federal Institute of Education, Science and Technology (IFPI), Campus Teresina Central, Teresina 64001-270, Piauí, Brazil; ⊥ Biotechnology Research Center, State University of Piauí, Teresina 64003-120, Piauí, Brazil; # Department of Physics, UFPI−Federal University of Piauí, Teresina 64049-550, Piauí, Brazil

**Keywords:** Hydroxyapatite, laponite, 3D printing, bone repair, emergency cases

## Abstract

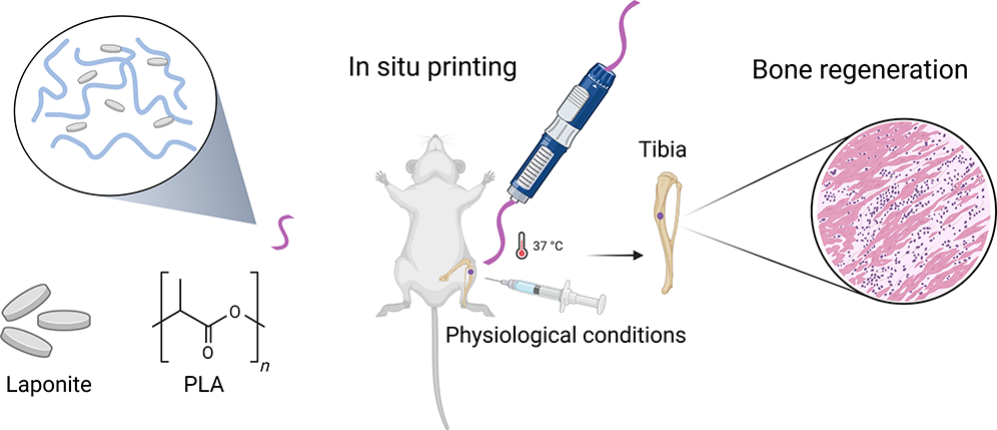

*In situ* 3D printing is attractive for the direct repair of bone defects in underdeveloped countries and in emergency situations. So far, the lack of an interesting method to produce filament using FDA-approved biopolymers and nanoceramics combined with a portable strategy limits the use of in situ 3D printing. Herein, we investigated the osseointegration of new nanocomposite filaments based on polylactic acid (PLA), laponite (Lap), and hydroxyapatite (Hap) printed directly at the site of the bone defect in rats using a portable 3D printer. The filaments were produced using a single-screw extruder (L/D = 26), without the addition of solvents that can promote the toxicity of the materials. *In vitro* performance was evaluated in the cell differentiation process with mesenchymal stem cells (MSC) by an alkaline phosphatase activity test and visualization of mineralization nodules; a cell viability test and total protein dosage were performed to evaluate cytotoxicity. For the *in vivo* analysis, the PLA/Lap composite filaments with a diameter of 1.75 mm were printed directly into bone defects of Wistar rats using a commercially available portable 3D printer. Based on the *in vitro* and *in vivo* results, the *in situ* 3D printing technique followed by rapid cooling proved to be promising for bone tissue engineering. The absence of fibrous encapsulation and inflammatory processes became a good indicator of effectiveness in terms of biocompatibility parameters and bone tissue formation, and the use of the portable 3D printer showed a significant advantage in the application of this material by in situ printing.

## Introduction

The shortage of bone implants is one of the biggest problems in underdeveloped countries, where thousands of people die every day due to the economic situation and lack of access to medical care and emergency rooms.^1,2^ The development of strategies to improve access, affordability and awareness to reduce the mortality rate has been deemed urgent.^3^ To address this problem, *in situ* 3D printing technologies have been developed to enable bone fillings outside the hospital and in emergencies.^4,5^ For example, physicians/surgeons could utilize *in situ* 3D printing technology, where a portable pen can print sterile filaments with bioresorbable, FDA-approved biopolymers (e.g., polylactic acid, PLA)^6^ with micro- and bioactive nanoceramics (e.g., hydroxyapatite, Hap, and nanosilicate, Lap)^7^ to perform treatments on people affected by fractures and unable to move in these cities.

Although the use of PLA-based nanocomposites with Hap and nanosilicate (e.g., Laponite, Lap) has been well researched over the past decade,^8,9^ it represents an innovation (especially in relation to the use of Lap) in bone tissue engineering (BTE). This is due to the unique properties that trigger specific biological responses, such as the ability to induce osteogenic differentiation of cells in the absence of osteoinductive factors^10,11^ and the ability to transport biochemical factors and bioabsorption of ions such as Ca, P, Si and Mg contained in Hap and Lap.^12,13^ Various approaches are currently being developed for the production and printing of materials for biomedical applications.^14−17^

Among these, 3D printing and bioprinting have attracted attention due to their ability to print materials with a high level of structural detail.^18^ The application of this technique again requires the use of predefined images for printing, which makes its application to bone defects a challenge for BTE, considering that features such as depth and size of the defect depend on uncontrollable factors in most cases.^19^ There are other limitations to the clinical applications of 3D printing/bioprinting, such as (i) the precision in the local surgical environment; (ii) the bioink requirements related to the low cell concentration and low viscosity of the bioink; (iii) the difficulty in controlling the extrusion pressure temperatures for local implantation (which is around 36.5 °C); (iv) the material thickness, which is directly related to the hydrogels; (v) the high costs associated with real monitoring (vi) and the slow response time in clinical emergencies, the specific knowledge required for the application and adequate infrastructure for this technique, sophisticated technologies with specialized expertise and trained personnel (not possible outside the hospital and in emergency interventions)^20,21^ are extremely poor and have limited access) and in emergencies where access to the hospital environment is not possible is extremely important.

Despite these challenges, *in situ* 3D printing is a rapidly developing field with ongoing research to solve the problems described. The advantages of *in situ* 3D printing compared to 3D printing are numerous. To better understand the advantages, the following points are described: (i) *in situ* 3D printing has gained notoriety as an innovative alternative for the treatment of tissue defects due to potential clinical applications;^19^ (ii) the concept of *in situ* 3D printing essentially refers to a bioink or biomaterial that is 3D printed directly at the fracture site;^22^ (iii) the use of portable 3D printers has gained visibility as they are portable for remote locations where healthcare is unsafe, inexpensive, easy to transport compared to traditional 3D printers and do not require specialized software for printing.^23^ (iv) the lack of bioinks that meet the specific and exclusive requirements of in situ bioprinting;^24^ (v) difficulties related to the temperatures for extrusion printing of biopolymers, where *in situ* 3D printing involves the constant temperature of the patient (37 °C) in addition to blood and other factors, unlike *in vitro* printing where there is an environment with controlled standards; (vi) to achieve adequate stability and production of hydrogel-based bioinks; (vii) the bioinks used for in situ printing must have properties that enable their release, transportation and reprocessing.

Following the described advantages of *in situ* 3D printing, we have developed composite filaments based on PLA, Lap and Hap by single-screw extrusion (to be used as filaments for clinical applications). A particularly innovative aspect of this work lies in the *in situ* 3D printing process using a portable 3D printer by extrusion on a model of bone defects combined with an alternative for rapid cooling immediately after *in situ* 3D printing, where the defect can be quickly filled. This model is intended for future emergency use in bone defects. In the search for a suitable and innovative composition for BTE, PLA + Lap composite filaments proved to be advantageous. Our strategy led to promising results in terms of alkaline phosphatase activity, cell adhesion and viability, and *in vivo* osseointegration which are favorable for *in situ* 3D printing.

## Materials and Methods

### Materials

Poly(lactic acid) (Nature Works, Ingeo 2003 D), Laponite (Lap, LAPONITE XLG-XR) provided by BYK USA INC XLG, Hydroxyapatite (Hap, provided by GRAPHEN TECHNOLOGY – R&D LABORATORY), 3D printing pen (MAXBROTHERS, China).

### Preparation of PLA and Bioactive Ceramics-Based Filaments

The filaments were produced using polymer extrusion technology. The proportions of each material used to define the study groups are described in Table 1.

**Table 1:** Sample Designation and Proportions Used^a^

Groups	PLA	Hap	Lap
P	100	0	0
PL	100	0	5.0
PH	100	5.0	0
PLH	100	2.5	5.0
PHL	100	5.0	2.5

a PCR = Parts per hundred of resin.

The PLA used in the study was predried in an oven at 60 °C for 8 h. The ceramics used were dried on a hot plate at 150 °C for 4 h. This experimental step aimed to remove any moisture that might be present in the materials and that could lead to the formation of bubbles in the polymer matrix during extrusion due to water evaporation. After drying, the materials were premixed in a beaker prior to the extrusion process.

The preparation and obtaining of the filaments were based on previous studies^25,26^ with modifications, A single-screw extruder (L/D = 26) model AX-16 from AX Plastics was used. The temperature stations were previously set to 170 °C, 180 °C, and 185 °C for zones 1, 2, and 3, respectively, with screw rotation maintained at 50 rpm.

The extruded material was cooled in water, and during the aforementioned processing, the filament diameters were controlled using a system of traction rollers equipped with a dial gauge and cooling by coolers, both from the Filmaq3D brand (Curitiba - PR). The parameters were adjusted according to initial tests to obtain continuous filaments with a diameter of 1.75 mm ±0.1.

### Characterizations

The morphology of the obtained filaments was studied by scanning electron microscopy (SEM) (FEI/FEG-250) with an accelerating voltage of 1 to 10 kV, equipped with energy dispersion spectroscopy (EDS) using SDD (silicon drift detectors), Ametek brand, model HX-1001, Apollo X-SDD detector, and the elemental composition was analyzed by energy dispersion spectroscopy (EDS). The thermal stability was analyzed by derivative thermogravimetry (DTG) of the thermogravimetric curve (TGA) using a TGA-51H device with a heating rate of 10 °C min-1 under a nitrogen atmosphere at a temperature of 800 °C. Differential scanning calorimetry (DSC) analysis was performed using a DSC-60 device to better investigate the temperatures and heat flows associated with the physical transitions of the materials.

### *In Vitro* Assays

*In vitro* tests were carried out in accordance with ethical principles adopted by the National Council for Control of Animal Experimentation (CONCEA) and received approval from the local Ethics Committee (Registration N^o^. 12/2020).

### Cell Isolation

The mesenchymal stem cells (MSCs) were obtained from the thighs of nine Wistar rats (*Rattus norvegicus*). After cleaning the femurs under a laminar flow hood, the bone marrow cells were isolated and seeded in 250 mL and 75 cm^2^ cell culture flasks (TPP, Biosystems, Curitiba, Brazil) containing essential alpha-MEM culture medium (Gibco) supplemented with 10% fetal bovine serum (FBS) (LGC Technology, Campinas, Brazil) and gentamicin (500 μg/mL) (Gibco). The cells were then incubated at 37 °C in a humidified atmosphere with 5% carbon dioxide (CO_2_). The culture medium was changed every 3 days and the development of the culture was assessed by inverse phase microscopy (Carl Zeiss - Axiovert 40C, Germany). After confluence, cells were enzymatically released and plated at a density of 1 × 10^4^ viable cells per well in a 96-well microplate (TPP, Biosystems, Curitiba, Brazil) as previously described.^27^

Before plating, the samples were weighed and sterilized under ultraviolet light and then placed in the wells. Osteogenic culture medium was added to the plate and changed every 48 h. After completion of these steps, all plates were incubated at 37 °C in a 5% CO_2_ atmosphere and maintained under these conditions until the time of testing. All tests were performed according to the ISO 10993–5 standard.

### Cell Adhesion

After 3 days of culture, cell morphology was analyzed by field emission scanning electron microscopy (FE-SEM) (Zeiss - EVO MA10, Carl Zeiss Pvt. Ltd., Oberkochen, Germany) at the Laboratory of Dental Materials – ICT UNESP. The samples with cells were fixed with 4% paraformaldehyde and dehydrated with ethanol. The samples were then coated with a thin layer of gold using a sputter coating system.

### Cell Viability

After the 3-day period, a quantitative evaluation of the living cells was performed after exposure to an MTT solution [3-(4,5-dimethylthiazol-2-yl)-2,5-diphenyltetrazolium bromide] (Sigma-Aldrich). The formazan crystals were dissolved by adding dimethyl sulfoxide (DMSO, Sigma-Aldrich) to each well. The plates were shaken at room temperature, and after dissolution of the crystals, the absorbance was measured spectrophotometrically at 570 nm (Micronal AJX 1900, Sao Paulo, Brazil). The results were expressed by converting the absorbance of the control to 100% viability.

### Determination of Protein Content and Alkaline Phosphatase Assays during MSC Differentiation

The total protein content was calculated over a period of 7 days to evaluate the interaction of the material in cellular protein production. The absorbance was measured spectrophotometrically at 680 nm (Micronal AJX 1900, Sao Paulo, Brazil). For the analysis of alkaline phosphatase (ALP) activity, it was determined using a specific kit that analyzes the thymolphthalein release reaction by hydrolysis of the thymolphthalein substrate. This procedure was carried out according to the manufacturer’s instructions (Labtest Diagnóstica, Minas Gerais, Brazil) over the same time period as the total protein, using the same lysates. The absorbance was measured with a spectrophotometer (Micronal AJX 1900, Sao Paulo, Brazil) at 590 nm.

### Mineralization Nodule Formation

After 14 days of culture, mineralization nodule formation was evaluated by staining with 2% Alizarin Red S (Sigma-Aldrich, St. Louis, United States), pH 4.2. The red dye from Alizarin Red S stains calcium-rich areas. Mineralization nodule formation was observed under an optical microscope (Axio Observer A1, Carl Zeiss, Germany)

### *In vivo* Analysis Using *In Situ* 3D Printing

*In vivo* tests were carried out in accordance with ethical principles adopted by the CONCEA and received approval from the State University of Piaui Ethics Committee (Registration N^o^. 016195/2022-9). Bone defects with a size of 5.4 mm were created with a 2.7 mm trephine cutter, resulting in an adjacent cavity of 2.7 × 2 = 5.4 mm. For the creation of this defect, a constant torque of 45N, a speed of 45,000 rpm and copious irrigation (70% setting of the electric motor) with saline were established to ensure the viability of bone regeneration. After the defect was created, PLA/Lap filaments with a diameter of 1.72 mm were printed using a portable 3D printer. Immediately after printing, saline was added for immediate cooling of the site (temperature around of 36.5 °C). The animals were sacrificed 30 days after lesion induction by an overdose of sodium thiopental (150 mg/kg). The bone segment containing the defect was removed, fixed in buffered formalin for 48 h and decalcified with EDTA (ethylenediaminetetraacetic acid), 10% w/v, pH 7.2. After decalcification, each specimen was cut longitudinally and the fragments were dehydrated in an automatic tissue processor (PT05 TS Luptec, São Paulo, Brazil) through a series of alcoholic solutions of increasing concentration and treated with xylene. After embedding in kerosene, histologic sections with a thickness of 5 μm were prepared using a rotary microtome (MRP09 Luptec, São Paulo, Brazil), which were then stained with hematoxylin and eosin (H.E.). The histological preparations were qualitatively analyzed with a trinocular light microscope (Olympus CX31, Japan) and photographed with a digital camera connected to a computer (Moticam WiFi X, MoticMicroscopes, Richmond, VA, USA).

### Statistical Analysis

All statistically analyzed tests were first subjected to the Kolmogorov–Smirnov normality test, and homogeneity of results was observed (*p* > 0.05). Data from the in vitro assays were analyzed using a two-way ANOVA (*p* < 0.05), n = 5, adjusting for the variables of time and biomaterial analyzed, and data were presented as mean ± standard deviation. When necessary, they were subjected to a Tukey’s post hoc test. All tests were performed using GraphPad Prism 7.0 software (GraphPad Software, San Diego, CA, USA). A significance level of 5% and a confidence interval of 95% were assumed for all statistical tests.

## Results and Discussion

**Figure 1:**
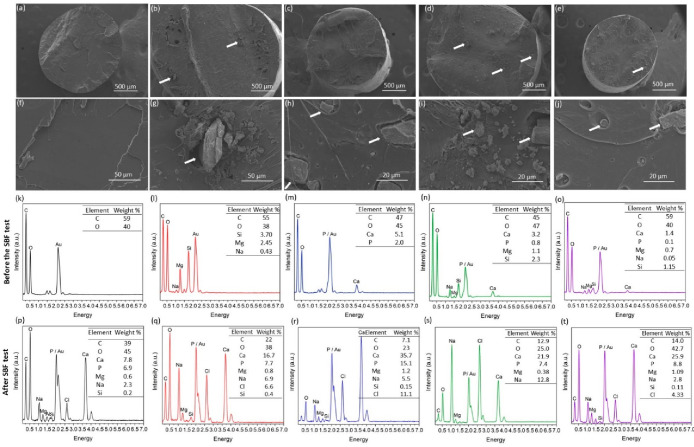
Scanning electron microscopy analysis of the obtained filaments for the (a, f) P, (b, g) PL, (c, h) PH, (d, i) PLH, and (e, j) PHL groups. (k–o) Graphs referring to Energy Dispersive Spectroscopy analysis before treatment with SBF solution. (p–t) Graphs referring to Energy Dispersive Spectroscopy analysis after 7 days of treatment with **SBF** solution, respectively for the **P**, **PL**, **PH**, **PLH**, and **PHL** groups. Clusters of nanoceramics incorporated into the PLA polymer matrix (white arrows).

Figure 1 shows the scanning electron images of the filaments obtained by the extrusion technique. The successive incorporation of the nanoceramics into the polymer matrix led to few morphological changes on the surface of the materials. In general, the filaments exhibited a rough surface in their macroscopic form, in contrast to group P, Figure 1(a–f), in which no charge was incorporated into the matrix. This property of the groups (PL, PH, PLH, PHL) is due to the addition of charges to the PLA polymer matrix. Figure 1(g–j) shows that the nanoceramics were not only incorporated on the surface of the material, but also in its interior. Similar results regarding the distribution of the incorporated materials were found in studies by.^28,29^ The polymer melt during the extrusion process together with the addition of ceramics promotes their agglomeration in the polymer matrix, as these particles are not soluble.^30^

Energy dispersive X-ray spectroscopy (EDS) was carried out at various points on the thread, on the surface and in cross-section. Figure 1(m–o) confirm the presence of peaks associated with calcium (Ca) and phosphorus (P) atoms, as shown in, present in the nHap structure. Peaks of silicon (Si), magnesium (Mg) and sodium (Na) were also identified, confirming the incorporation of Lap nanoclay into the PLA matrix, as shown in Figure 1 (i–o).^31^

The results of the EDS analysis complement the SEM analysis in Figure 1(a–j). They confirm the presence of chemical elements in the ceramic nanoparticles incorporated in the polymer and their agglomerated distribution in the matrix, as shown in Figure 1(g–j) This result can be attributed to the extrusion technique chosen for processing.

We can observe that the groups with a higher Lap content in their composition (PL and PLH) showed a greater tendency to particle agglomeration in the polymer matrix compared to the other study groups, as shown in Supplementary Figure S1(b) and (d), while the groups in which Hap was incorporated tended to disperse more uniformly after extrusion, as shown in Supplementary Figure S1(c) and (e). This observed behavior can be attributed to intrinsic properties of Lap, such as its tendency to form stacks due to electrostatic interactions with sodium ions present in its structure,^12^ which could directly affect the mechanical properties due to the formation of stress points resulting from the agglomeration of the fillers in the polymer matrix. These results are confirmed by the mechanical analysis in Supplementary Figure S4(a).

Figure 1(p–t) shows the graphs relating to the energy dispersion spectroscopy analysis after 7 days of treatment with SBF solution. Compared to the results shown in Figure 1(i–o), there is a clear increase in the percentage of chemical elements such as calcium (Ca) and phosphorus (P). This confirms the formation of apatite on the surface of the material, indicating its ability to adsorb calcium and other elements necessary for the formation of bone tissue. The formation of apatite on the surface of the material provides specialized cells with a structure that gives them support and consequently allows complete differentiation.^32^

**Figure 2:**
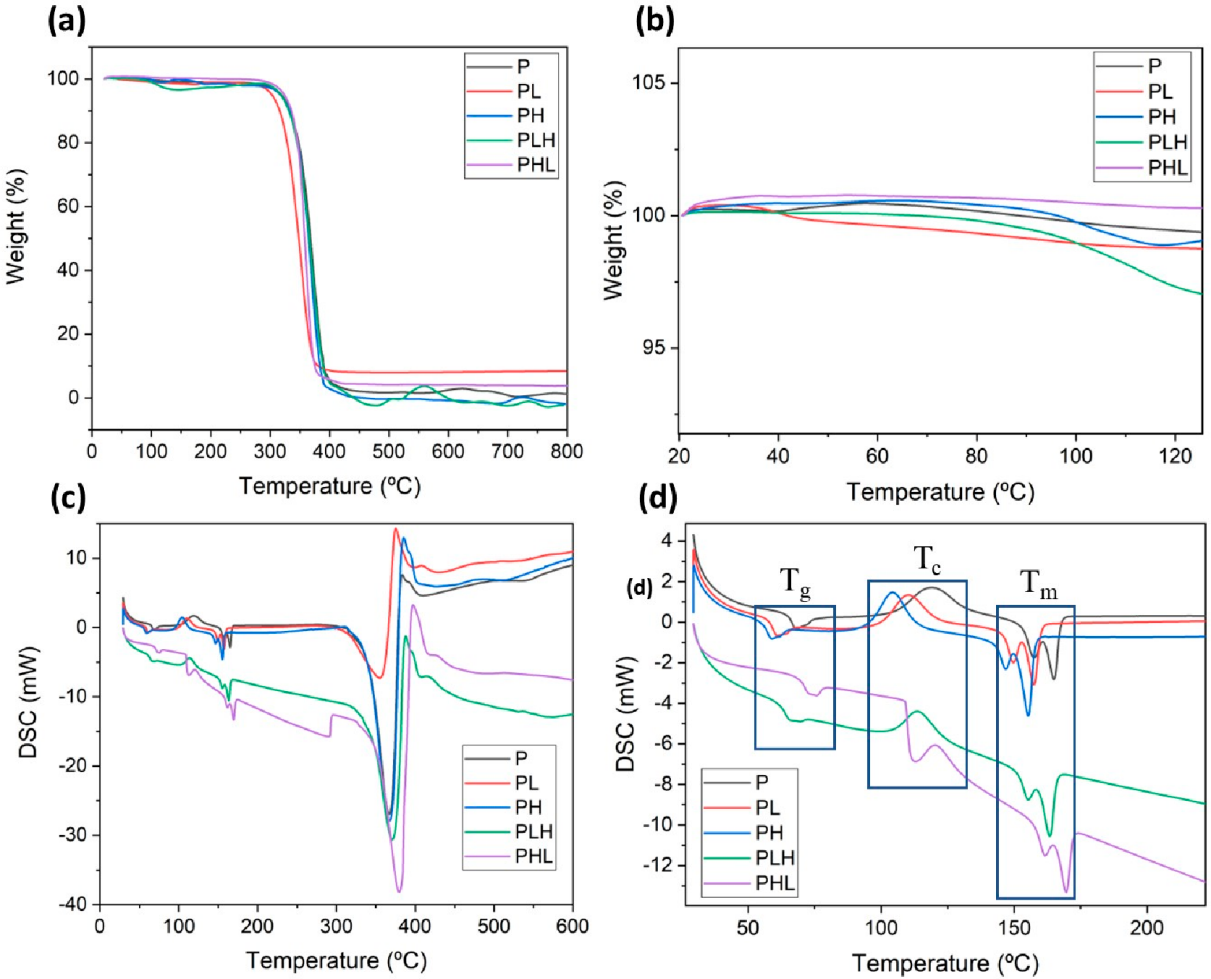
(a, b) Thermal analysis curve of thermogravimetry (TGA), (c, d) Differential Scanning Calorimetry (DSC). (b) Identification of Glass Transition (Tg), Crystallization (Tc), and Melting (Tm) processes.

In general, the other groups showed similar results with respect to the main stages of thermal decomposition compared to the control group in which the filament was made only from PLA. The main decomposition temperatures (initial time) were between 310 and 335 °C, with the materials initially showing a mass reduction of about 2% (Figure 2a). This was not a significant loss that could be attributed solely to the decomposition of volatile elements. It is also important to note that the PLH group exhibited two decomposition phases at temperatures of 150 and 300 °C (Figure 2a).

In the final decomposition, the materials showed slight differences compared to the control group (Figure 2a): (final temperature) 720 °C for group P, at which complete degradation was observed; PL 800 °C, at which 92% mass loss was observed; PH and PLH (final temperature) 400 °C, at which complete degradation of the material was observed; and PHL 800 °C, at which 96% degradation was observed. Comparing the results with literature studies on polymers for 3D printing techniques based on extrusion, it can be seen that the initial degradation temperature (*T*_initial_ = 330 °C) for group P (Figure 2b) is close to the values obtained in the present study.^33^ In studies by Carrasco et al.^34^ a 95% reduction in the initial mass of P was observed at 377 °C, which is comparable to the results of this study.

The derivative of the thermogravimetric curve (DTG) showed peaks corresponding to the points of maximum mass change with respect to temperature, indicating that all study groups reached similar temperatures in the range of 350 to 365 °C, as shown in Supplementary Figure S2. The results of TG and DTG indicate that there was little or no chemical interaction between PLA and the incorporated ceramics, which can be attributed to the processing method chosen, where temperature was the most important element.

The curves obtained from the analysis of the differential calorimetry, as shown in Figure 2(d), exhibit three typical patterns found in semicrystalline thermoplastics like PLA. The first represents the heat flow at the glass transition temperature (Tg), the second, an exothermic curve originating from a cold crystallization process, and finally, an endothermic curve related to melting (Tm). The Tg of groups PL (53 °C) and PH (50 °C) showed a slight reduction compared to group P (60 °C). The crystallization region of groups PL (101 °C) and PH (97 °C) also exhibited a slight reduction compared to group P (111 °C) (Figure 2d). The curves obtained from the differential calorimetry analysis, as shown in Figure 2(d), show three typical patterns found in semicrystalline thermoplastics such as PLA. The first represents the heat flow at the glass transition temperature (Tg), the second an exothermic curve resulting from a cold crystallization process, and finally an endothermic curve related to melting (Tm). The Tg of groups PL (53 °C) and PH (50 °C) was slightly reduced compared to group P (60 °C). The crystallization range of groups PL (101 °C) and PH (97 °C) also showed a slight reduction compared to group P (111 °C) (Figure 2(d)).

For the PH group, the decrease in melting temperature and glass transition area is consistent and amounts to about 10 °C at a nanofiller concentration of 5%. The PL group shows a decrease of about 7 °C for the same concentration of nanofillers. The presence of two peaks in the melting temperature of the materials has already been observed in previous studies and is justified by the formation of several crystalline states, a result of the thermal processing of the material during filament fabrication, Figure 2 (d). This behavior is also influenced by the presence of inorganic additives such as hydroxyapatite and Lap as well as impurities in the PLA polymer matrix.^35^ The presence of a cold crystallization region, characteristic of the exothermic process of self-nucleation of crystalline phases that occurs after the glass transition temperature, follows the same pattern of a slight decrease in groups with nanofillers compared to group P. This is a consequence of solid-state transitions between different crystalline states.^36^

The groups resulting from the mixture of more than one type of ceramic nanofiller show different patterns. The PLH group with a higher proportion of Lap (5%) than Hap (2.5%) shows an increase in Tg and Tf of about 5 °C compared to the PL group containing only laponite. The PHL group shows a different trend from the others, with an increase in Tg and Tf of about 5 °C compared to the P group, Figure 2(d) In the region where cold crystallization should occur, the PHL group undergoes a second glass transition, indicating the absence of autonucleation, which is replaced by the appearance of an amorphous phase in a flexible state of the polymer.^33^

As shown in Figure S4(a) Supporting Information, the P group exhibited a tensile strength of 58.2 MPa, which underlines the acceptable mechanical properties of PLA compared to.^37^ However, for the PL group, this strength decreased to 16.6 MPa, indicating that Lap introduced weak points into the composite, which is evident from the irregular filament formation after extrusion (Figure S4(c)).^38,39^ The incorporation of Hap into the PH group resulted in a tensile strength of 41.7 MPa, indicating a potential mechanical improvement depending on the interaction with PLA. Tensile strengths of 37.17 and 64.3 MPa were observed for the PLH and PHL groups, respectively, suggesting a synergistic effect between the two reinforcements. Effective dispersion and interaction between PLA and the reinforcing particles are crucial. The lower amount of Lap may have minimized agglomeration, while the higher amount of Hap provided significant reinforcement.

The neat PLA exhibited an elastic modulus of 1750 MPa, as seen in Figure S4(b), which is consistent with results in the literature.^38^ With the addition of Lap, the elastic modulus decreased to 600 MPa, suggesting that it did not effectively contribute to the stiffness of the material and may have led to the formation of sharp edges, as seen in Figure S4(b), resulting in a more flexible and less stiff composite. The incorporation of Hap resulted in a modulus of elasticity of 1600 MPa, which is close to that of pure PLA. Hap helped to maintain the stiffness of the composite, although the slight reduction compared to pure PLA suggests that the dispersion or interface between Hap and PLA may not be ideal. The PLH group exhibited a modulus of 1700 MPa, suggesting that Hap helped to maintain stiffness despite laponite. The PHL group achieved a modulus of 2200 MPa, indicating strong dispersion and interaction between PLA and the reinforcing materials. Improving the interfacial interactions between PLA and reinforcements could further increase the stiffness of the material.

Studies on bone cell activity and differentiation are of utmost importance for proper osseointegration of *in vivo* implants.^40^ Here, we performed a study to evaluate the influence of produced filaments on the differentiation process of MSC into bone tissue cells through ALP activity, which is an indicator of osteoblast activity,^41^ and staining with alizarin red.

**Figure 3:**
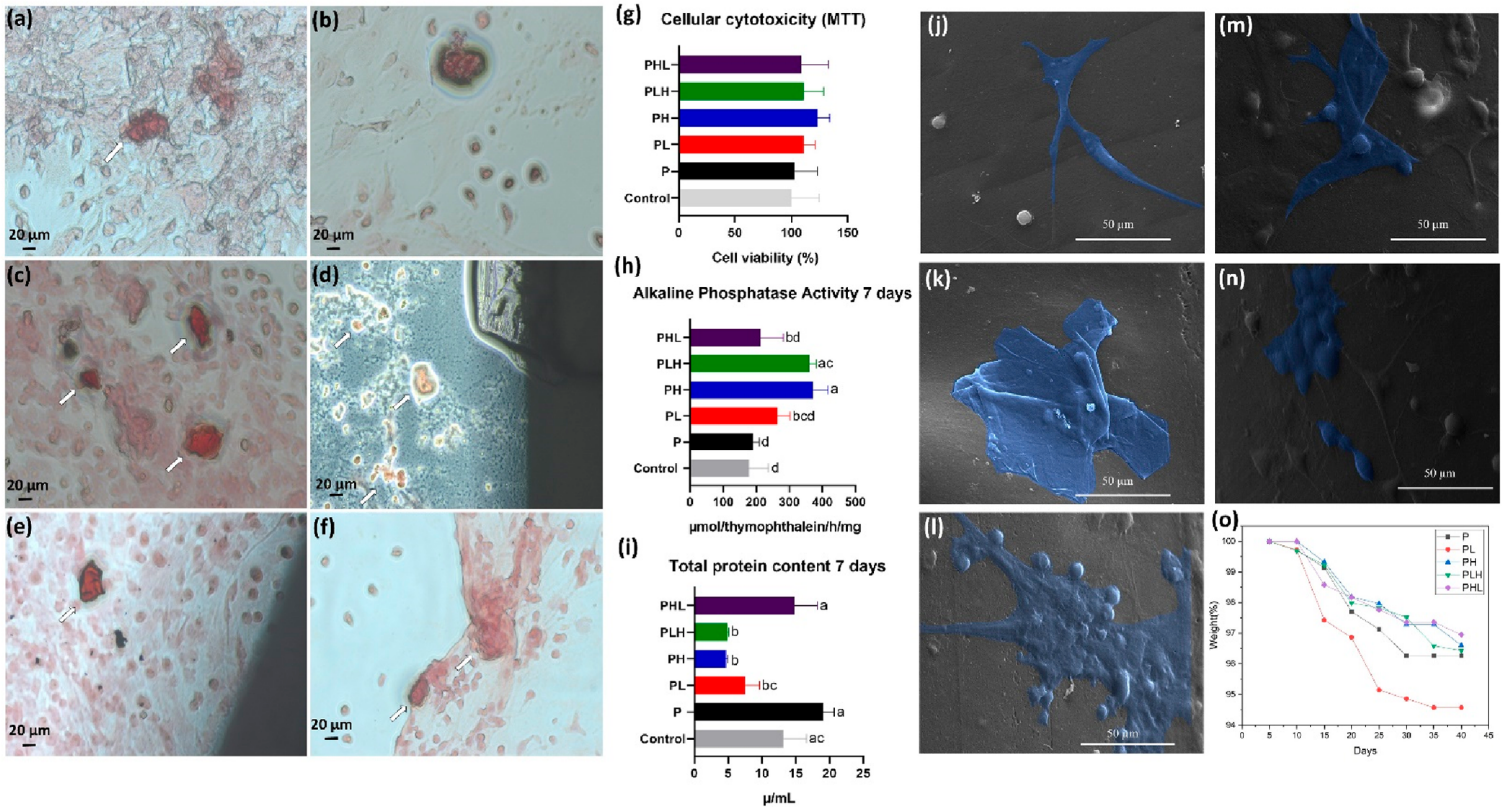
Analysis of in vitro results, (a–f) Alizarin red staining, respectively: (a) Control, (b) **P**, (c) **PL**, (d) **PH**, (e) **PLH**, (f) **PHL**, (g) Cellular Cytotoxicity, (h) Alkaline Phosphatase Activity at 7 days, (i) Total Protein Content at 7 days, (j–n) Cell Adhesion viewed by Scanning Electron Microscopy, respectively: (j) **P**, (k) **PL**, (l) **PH**, (m) **PLH**, (n) **PHL**. (o) Degradation test in **PBS**. In vitro data presented as mean value ± standard, *n* = 5, deviation analyzed by two-way ANOVA (*p* < 0.05), subjected to Tukey’s posthoc test.

Figure 3(a–f) show the formation of mineralization nodules in cell culture analyzed from alizarin red. Clearly, PL (Figure 3c), PH (Figure 3d) and PLH (Figure 3e) groups appear to have greater formation (indicated by the white arrows) compared to control (Figure 3a), P (Figure 3b), PH (Figure 3d) and PHL (Figure 3f) confirming the differentiation of the cells tested into osteoblasts. This result confirms the role of incorporated nanoceramics in the osteogenic differentiation process. Moreover, these results are consistent with the activity of alkaline phosphatase, which was higher in the respective groups, as shown in Figure 3h, as well as with the significant increase in the percentage of the chemical elements calcium (Ca) and phosphorus (P) after treatment with SBF, as shown in Figure 1q–s, confirming the formation of apatite on the surface of these materials. This indicates that the nanoceramics added to the polymer matrix influenced the formation of the mineralized matrix. It is important to note that in vitro studies are limited as they do not fully reflect the cellular interactions and structural properties of the tissue. Therefore, further studies are needed to better understand how the obtained materials interact with biological systems and the mechanisms of bone formation.^42^

As far as cell viability is concerned, the MTT test showed that there were no statistical differences between the groups (*p* > 0.05). All groups showed a cell viability of 100 or more on the third day of treatment, indicating the nontoxicity of the materials obtained. This factor favors their application in biological systems (Figure 3g). Similar studies involving the preparation of biopolymer-based filaments using calcium phosphates have shown comparable results in cell viability tests to assess the cytotoxicity of filaments, confirming the results of the present study. Bioceramics such as Hap shows good activity in osteoconductive processes, resorption and also facilitate protein fixation. Meanwhile, PLA has properties such as biocompatibility, nontoxicity and biodegradability and serves as a polymeric matrix for load delivery.^26^

The examination of total protein showed that the control groups, P and PHL, had no statistical differences between each other (*p* > 0.05) and that they had higher total protein levels on day 7, which were statistically different from those of all other groups (*p* < 0.05). On the other hand, the PH and PLH groups had lower levels and were statistically different from all groups (*p* < 0.05), except for the PL group. However, the PH and PLH groups did not present statistical differences between them (*p* > 0.05), as shown in Figure 3(i). Although a significant decrease in total protein content was observed, the results showed that all tested filaments similarly stimulated ALP activity, an important marker of osteogenic differentiation.^43^ This indicates that the mesenchymal cells used in the study were able to differentiate into osteoblasts when they encountered the filaments obtained.

The ALP activity test showed that the PL, PH and PLH groups had the highest alkaline phosphatase activity values: PL (264.9), PH (372.1) and PLH (360.0). The PH and PLH groups showed no statistical differences between them (*p* > 0.05) and showed higher values. On the other hand, the control and P groups showed lower values and were statistically different from the PH and PLH groups (*p* < 0.05) but did not show statistical differences with the PL and PHL groups (*p* > 0.05) (Figure 3h). Overall, all groups showed good results compared to the control group. However, the results of this test indicate that Lap had a positive effect on initiating the cellular differentiation process in certain tissues and is comparable to nanoceramics already used for BTE such as Hap. Similar results regarding the osteogenic bioactivity of Lap are found in studies by Mousa and colleagues,^44^ in which the effect on the differentiation process is due to the expression of genetic markers that are precursors of ALP. Other studies also show the positive effect of Lap on the osteogenic differentiation process depending on the concentration.^45^

After 3 days of cultivation, the samples were analyzed by SEM to detect the cellular interaction with the filaments produced, as shown in Figure 3 (J–n). In this analysis, the presence of adherent cells on the surface of the produced material was observed, indicating that all samples showed potential interaction with the cells. Compared to the control group (PLA), the other experimental groups exhibited a surface area indicative of higher adhesion. Similar results were found by^26^ in studies on the production of PLA-based filaments with biphasic calcium phosphates. However, it is important to emphasize that the mechanisms of cellular interactions in osteoblast anchorage, development and proliferation depend on various factors, such as topographic conditions, crystallite size and phosphate dissolution rate.

Studies dealing with the fabrication of osteoconductive filaments are significant considering that the fabrication of filaments by extrusion is already a well-established technique in additive manufacturing,^46,47^ which facilitates the scaling of these materials. In addition, the materials produced in the form of filaments in this study are characterized by their ease of use in clinical applications for in situ printing with portable 3D printers.

Degradation is one of the relevant factors that must be taken into account when applied in living systems, as materials degrade over time when they come into contact with biological fluids and are gradually replaced by the formation of new tissue.^48^ As shown in Figure 3(o), we observed that the group with the composition PLA + 5% Lap (PL) had a higher degradation rate in PBS solution compared to the other groups, with a degradation rate of approximately 5.2%. The degradation of laponite in biological media would lead to the release of products such as orthosilicic acid, which is found in blood plasma and has functions related to the formation of type I collagen.^49−51^ Therefore, for in vivo studies, it was decided to proceed only with the PLA + 5% Lap (PL) group, as it showed a higher degradation rate compared to the other groups in this study, satisfactory performance in terms of ALP activity, and good cell adhesion.

*In-situ* 3D printing of polymers can be challenging during intraoperative procedures due to properties such as the working temperature of thermoplastic polymers. To investigate the thermal behavior in surgical situations, we propose a new alternative for rapid cooling after in situ printing with saline. Using a thermal imaging camera, we observed an immediate decrease of 30 °C in the temperature of the polymer after printing, and it reached a temperature of 38.3 °C in 5 s after the addition of PBS. This shows superior results compared to the study conducted by,^6^ in which heat transfer analysis using polymers with nanocarriers in portable 3D printers showed thermal stability in 68 s, see Supplementary Figure S3 and Supplementary Video S1.

**Figure 4:**
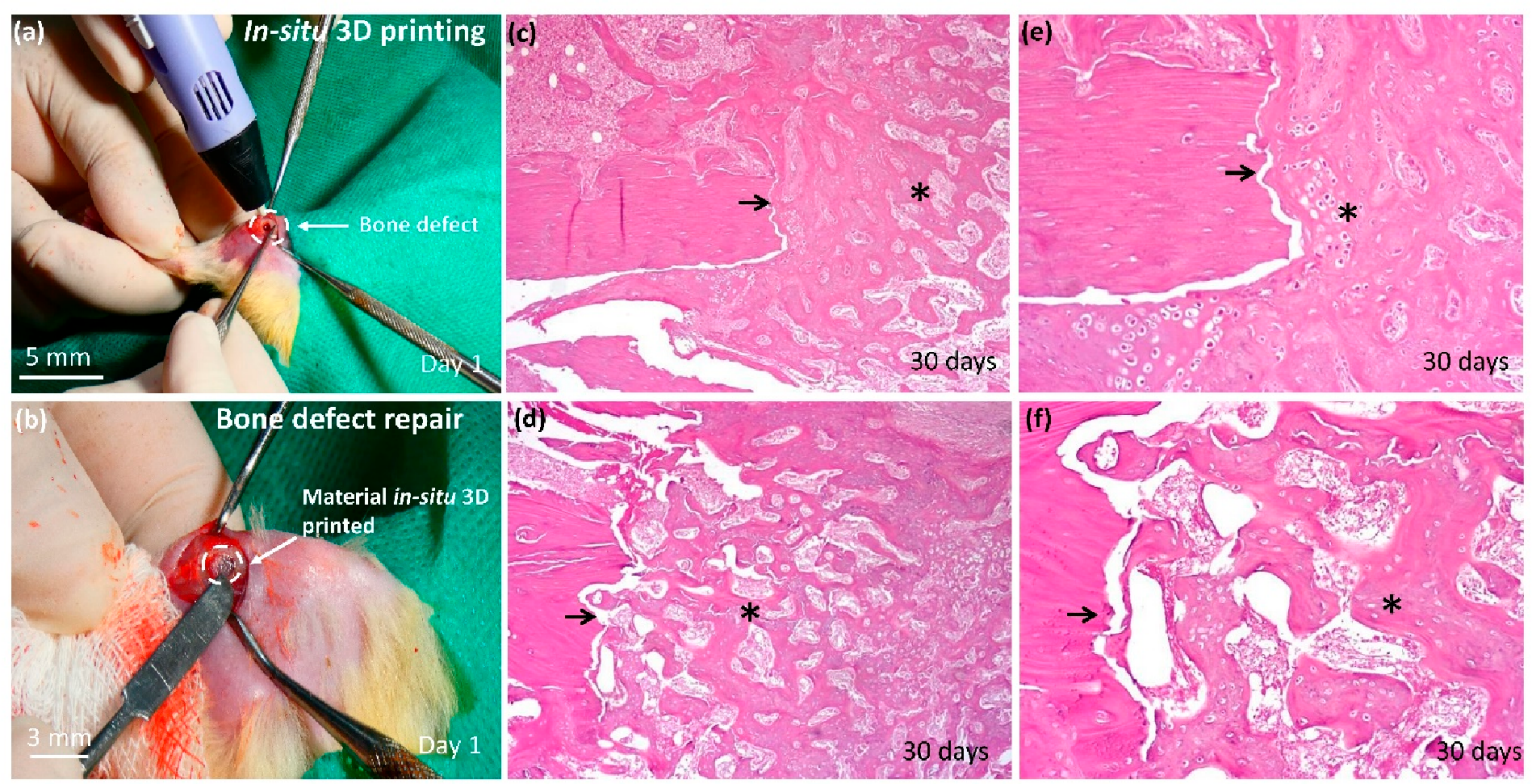
*In situ* printing and histological analysis. (a) Bone defect created for the study of the regenerative process. (b) Bone defect filled with biomaterial using the in situ printing technique with a 3D pen. (c) Histological analysis of the control group using hematoxylin and eosin (H&E) staining with magnification (40×). (d) Histological analysis of the PL group using hematoxylin and eosin (H&E) staining with magnification (100×). (e) Histological analysis of the control group using hematoxylin and eosin (H&E) staining with magnification (40×). (f) Histological analysis of the PL group using hematoxylin and eosin (H&E) staining with magnification (100×).

To demonstrate the applicability of the *in situ* 3D printing technique, PLA composite filaments containing 5% Lap (PL) with a diameter of 1.72 mm were printed for the first time using a commercially available portable 3D printer into bone defects with a diameter of 5.4 mm extending to the medullary canal, as shown in Figure 4(a). We can observe the filling and adhesion of the material to the defect, demonstrating the effectiveness of the technique in cases of immediate application, as shown in Figure 4(b). The use of the portable 3D printer facilitates the application and speed in the use of materials in harmful defects. The performance of the portable 3D printer can be better observed in Supplementary Video S2.

The descriptive histological analysis used as a reference point the dividing line between the pre-existing cortical bone with lamellar pattern and the newly formed tissue. This analysis was performed after 30 days, a period chosen based on previous studies describing the formation of organized bone trabeculae during this period.^52,53^ In both the control group and the PL group, the newly formed tissue filled the bone defect and extended to both the periosteum and the medullary region. This tissue consisted of interconnected trabeculae of varying thickness immersed in a loose extracellular matrix rich in fibroblasts. In the control group (Figure 4c and 4e), the trabeculae appeared thicker and irregular and contained numerous chondroid-like osteocytes. In the PL group (Figure 4d and 4f), the trabeculae were thinner and appeared histologically more mature than in the control group, with fewer chondroid-like osteocytes. In addition, fibrous encapsulation or inflammatory process was not evident at the bone-biomaterial interface. Bone trabeculae are distributed in areas exposed to high mechanical loading and play a role as an effective system to increase bone strength.^54^ During implantation, there is an interaction between the biomaterial and the immune system that triggers specific cellular and tissue reactions such as scarring, inflammatory reactions, and fibrous encapsulation of the implanted biomaterial.^55^ The lack of combination of fibrous encapsulation and inflammatory processes is a good indicator that demonstrates the efficiency of 3D implantation in situ, but also the rapid recovery of the implants that no malfunction occurred based on the histological analysis at the temperatures used for imprinting. This performance could also be due to the characteristics observed in the in vitro tests, such as high cell viability and ALP activity (Figure 3h). Similar results regarding the success of in situ extrusion printing applications have been reported in other studies.^6^ However, to date we have not identified any studies addressing in situ extrusion applications of polymers in models of bone tissue defects.

We have successfully developed a portable integrative bone repair strategy that can be used in underdeveloped countries and in emergency situations. This strategy is extremely forward-looking and may represent the future of *in situ* 3D printing. At the same time, we have developed a new nanocomposite based on an FDA-approved polymer and nanobioceramic that works *in vitro* and *in vivo*. The developed filaments can be easily sterilized, packaged, and transported in back pockets by doctors and dentists. At the same time, we can consider a portable pen: easy to handle, cheaper, low power consumption (for use in a portable battery) and extremely easy to find on the market. Then we can consider our strategy as the next generation of biomaterials that can be used by patients for newly occurring bone defects.

## Conclusion

We have succeeded in developing an *in situ* 3D printing strategy for the direct repair of bone defects in underdeveloped countries and in emergency situations. Filaments of PLA and Hap/Lap nanoceramics were successfully produced by extrusion and proved to be suitable for the production of this type of material. The applied processing had no significant influence on the thermal properties of the filaments, as thermogravimetric and differential scanning calorimetry analyzes showed. The EDS technique confirmed the integration of the nanoceramics into the polymer matrix of the filaments. All groups showed good cell viability, indicating noncytotoxicity by the MTT assay. In addition, the PL group showed adequate results in the analysis of alkaline phosphatase activity and mineralized matrix formation, indicating an interaction with the osteogenic differentiation process of mesenchymal stem cells, as well as a higher degradation rate compared to the other groups, so it was selected for *in vivo* application and proof of concept. Based on the *in vivo* results, the *in situ* 3D printing strategy showed promise for BTE, and the rapid cooling alternative showed favorable results in conjunction with *in situ* polymer extrusion. The absence of fibrous encapsulation and inflammatory processes became a good indicator of efficacy in terms of biocompatibility parameters and bone tissue formation.

## Supporting Information Available

EDS mapping collected from all analyzed groups; derivative thermogravimetry analysis (TGA/DTG); cooling curve after in situ 3D printing of PLA + Laponite (5%) using portable pen; mechanical tensile test of filaments (PDF)
Video S1, thermal stability in 68 s (MP4)
Video S2, performance of the portable 3D printer (MP4)
