# Supplementary material for: In Situ Printing of Polylactic Acid/Nanoceramic Filaments for the Repair of Bone Defects Using a Portable 3D Device
Source: ACS Appl Mater Interfaces. 2024 Jul 21;17(9):13135–45. doi: 10.1021/acsami.4c05232 (PMC11891858; doi:10.1021/acsami.4c05232)
Supplement: Supplementary file 3 — am4c05232_si_003.pdf [file am4c05232_si_003.pdf]

Supporting Information

In-situ printing of polylactic acid/nanoceramic filaments for the repair of bone defects using a portable 3D device

Guilherme Castro Brito[a], Gustavo Fernandes Sousa[a], Moises Virgens Santana[a], André Sales Aguiar Furtado[a], Millena de Cassia Sousa E Silva[a], Thiago Ferreira Candido Lima Verde [a], Renata Barbosa[b], Tatianny Soares Alves[b], Luana Marotta Reis Vasconcellos[c], Leonardo Alvares Sobral Silva[c], Vicente Galber Freitas Viana[d], José Figueredo-Silva[e], Antônio Luiz Martins Maia Filho[e], Fernanda Roberta Marciano[a,f] and Anderson Oliveira Lobo[a]*

[a] LIMAV—Interdisciplinary Laboratory for Advanced Materials, BioMatLab, Materials Science & Engineering Graduate Program, UFPI—Federal University of Piauí, Teresina 64049-550, PI, Brazil

[b] LAPCON— Laboratory of polymers and conjugated materials, Technology Center CT, Materials Science & Engineering Graduate Program, UFPI—Federal University of Piauí, Teresina, 64049-550, PI, Brazil

[c] Institute of Science and Technology, São Paulo State University (UNESP) 777 Eng. Francisco José Longo Avenue, São José dos Campos, 12245-000, SP, Brazil.

[d] Postgraduate Program in Materials Engineering, Federal Institute of Education, Science and Technology (IFPI), Campus Teresina Central, Teresina, PI, 64001-270, Brazil

[e] Biotechnology Research Center - State University of Piauí, Teresina, PI, CEP 64003-120, Brazil.

[f] Department of Physics, UFPI—Federal University of Piauí, Teresina, 64049-550, PI, Brazil.

*lobo.aol@gmail.com and lobo@ufpi.edu.br

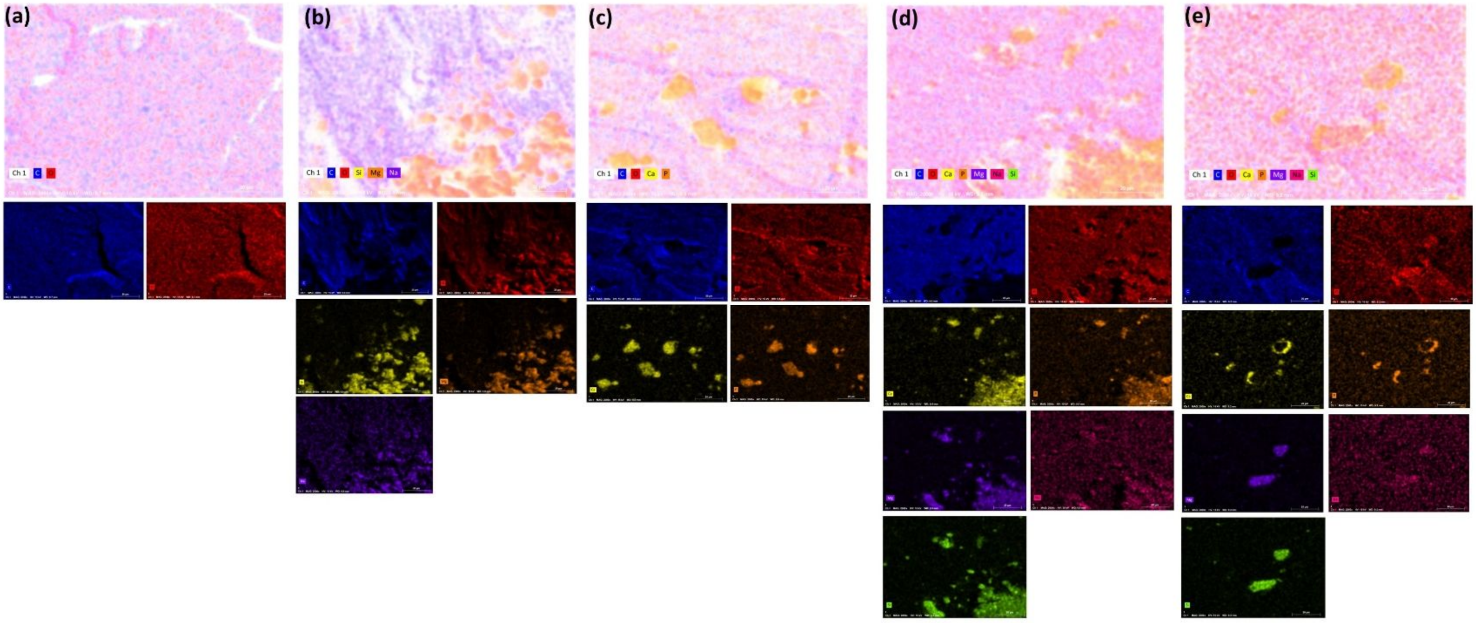

Figure S1. EDS mapping collected from all analyzed groups P (a), PL (b), PH (c), PLH (d), PHL (e).

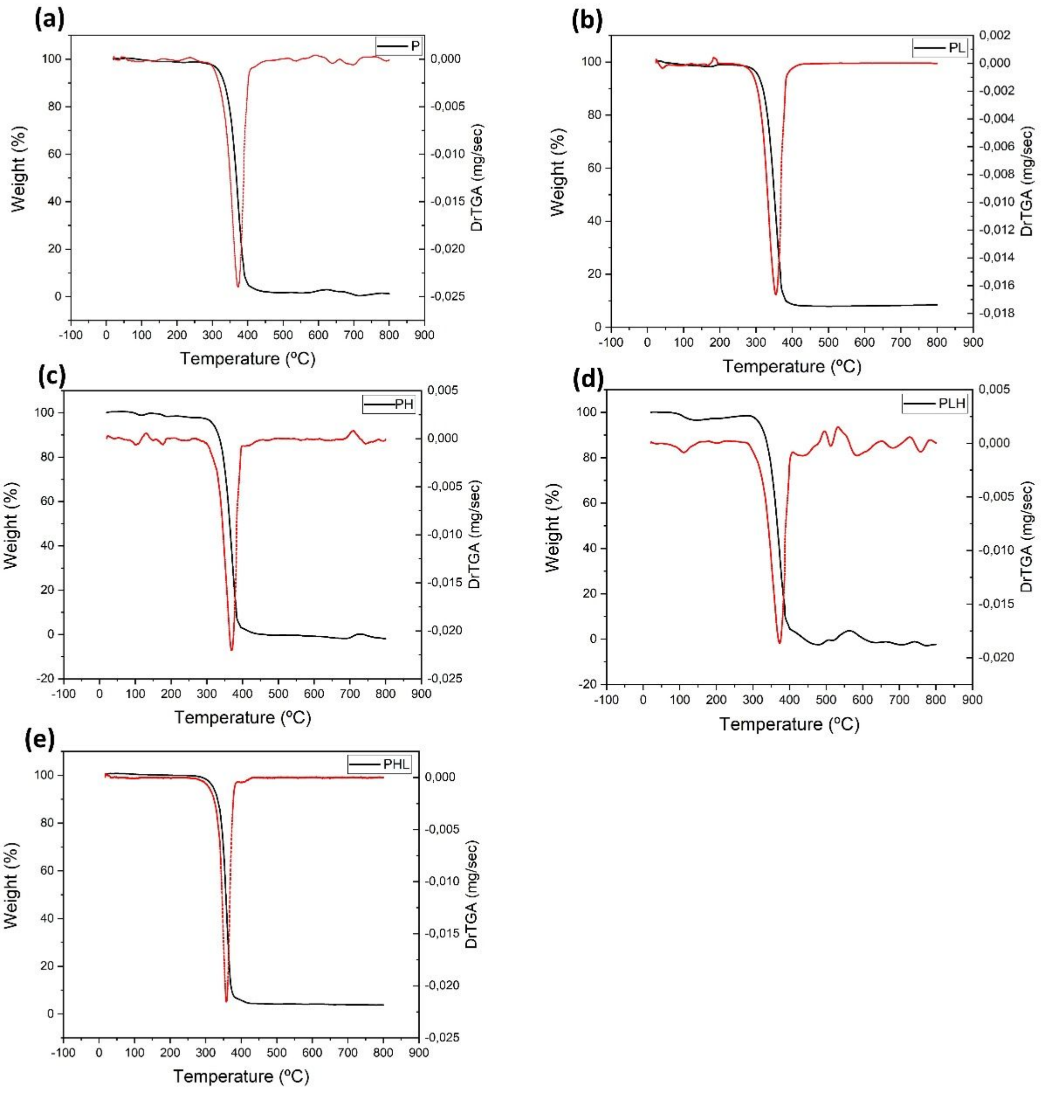

Figure S2. Derivative thermogravimetry analysis (TGA/DTG): (a) P, (b) PL, (c) PH (c), (d) PLH, (e) PHL

Analysis of the derivative of the thermogravimetric curve (DTG) revealed peaks consistent with the cases of maximum mass change with respect to temperature. This means that all study groups reached similar temperatures in the range of 350 to 365 °C

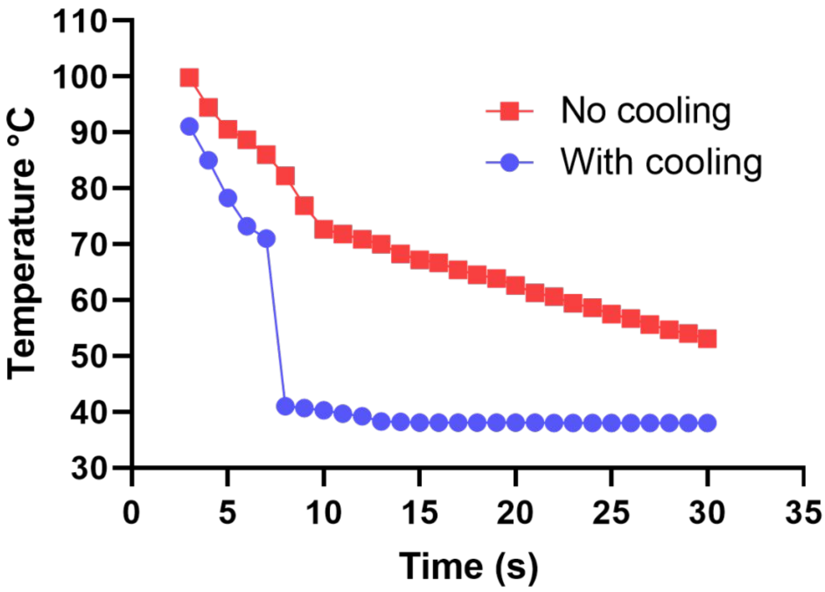

Figure S3. Cooling curve after *in-situ* 3D printing of PLA + Laponite (5%) using portable pen.

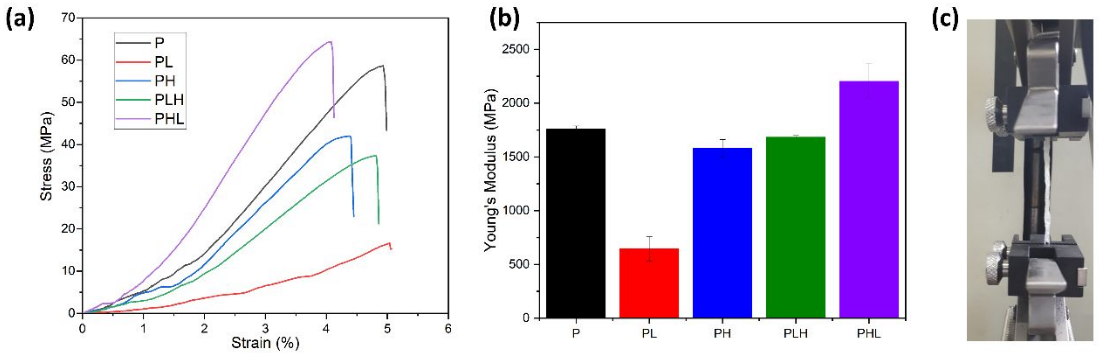

Figure S4. Mechanical tensile test of filaments. (a) – Stress vs. strain graph; (b) – Young's Modulus; (c) – Example of the fixed filament to the grips of the equipment during the mechanical test (PL group)
